# Combinatorial synthesis and screening of cancer cell-specific nanomedicines targeted via phage fusion proteins

**DOI:** 10.3389/fmicb.2015.00628

**Published:** 2015-06-23

**Authors:** James W. Gillespie, Amanda L. Gross, Anatoliy T. Puzyrev, Deepa Bedi, Valery A. Petrenko

**Affiliations:** Department of Pathobiology, College of Veterinary Medicine, Auburn UniversityAuburn, AL, USA

**Keywords:** phage display, targeted nanomedicines, breast cancer, pancreatic cancer, liposomal drug delivery, doxorubicin

## Abstract

Active tumor targeting of nanomedicines has recently shown significant improvements in the therapeutic activity of currently existing drug delivery systems, such as liposomal doxorubicin (Doxil/Caelyx/Lipodox). Previously, we have shown that isolated pVIII major coat proteins of the fd-tet filamentous phage vector, containing cancer cell-specific peptide fusions at their N-terminus, can be used as active targeting ligands in a liposomal doxorubicin delivery system *in vitro* and *in vivo*. Here, we show a novel major coat protein isolation procedure in 2-propanol that allows spontaneous incorporation of the hydrophobic protein core into preformed liposomal doxorubicin with minimal damage or drug loss while still retaining the targeting ligand exposed for cell-specific targeting. Using a panel of 12 structurally unique ligands with specificity toward breast, lung, and/or pancreatic cancer, we showed the feasibility of pVIII major coat proteins to significantly increase the throughput of targeting ligand screening in a common nanomedicine core. Phage protein-modified Lipodox samples showed an average doxorubicin recovery of 82.8% across all samples with 100% of protein incorporation in the correct orientation (N-terminus exposed). Following cytotoxicity screening in a doxorubicin-sensitive breast cancer line (MCF-7), three major groups of ligands were identified. Ligands showing the most improved cytotoxicity included: DMPGTVLP, ANGRPSMT, VNGRAEAP, and ANDVYLD showing a 25-fold improvement (*p* < 0.05) in toxicity. Similarly DGQYLGSQ, ETYNQPYL, and GSSEQLYL ligands with specificity toward a doxorubicin-insensitive pancreatic cancer line (PANC-1) showed significant increases in toxicity (2-fold; *p* < 0.05). Thus, we demonstrated proof-of-concept that pVIII major coat proteins can be screened in significantly higher throughput to identify novel ligands displaying improved therapeutic activity in a desired cancer phenotype.

## Introduction

Cancer remains the second leading cause of death in the United States across all age groups for both genders and continues to produce a significant burden on the healthcare system for improved patient outcomes with an improved quality of life in cancer survivors (Siegel et al., 2015). Treatment options include surgery, chemotherapy, radiation therapy or a combination of the three and are dependent on the tumor stage at initial diagnosis and the overall health/age of the patient. For example, breast cancer is commonly treated with surgery and radiation therapy (60%) in early stages (I or II) with the addition of chemotherapy in some cases (39%). However, in later stages (III or IV), chemotherapy is used significantly more often (74%) in a patient's treatment strategy (DeSantis et al., 2014). Tumor management with chemotherapy has shown to prevent tumor recurrence and prolong a patient's overall survival, but can also decrease the quality of life due to non-specific side effects associated with the high toxicity profile of many chemotherapy treatments (Early Breast Cancer Trialists' Collaborative Group, 2005; Riihimäki et al., 2012).

Pegylated liposomal doxorubicin (Doxil, Caelyx, or Lipodox) was introduced as the first FDA-approved nanomedicine composed of the commonly used anticancer drug doxorubicin encapsulated within the core of liposomes. Liposomal doxorubicin is the first nanomedicine approved for use in the clinic for a variety of cancer types. It showed significant improvements in circulation and bioavailability, and leads to overall reduction of tumor volume while significantly increasing the long-term survival of patients (Gabizon et al., 2003). Nanomedicines provide an increase in therapeutic activity by passively targeting their therapeutic payloads and using the leaky vasculature commonly associated with many tumors, a pathophysiological phenotype known as the enhanced permeability and retention (EPR) effect (Fang et al., 2011). It has been suggested extensively from pre-clinical models that active targeting of nanomedicines can provide an additional improvement in the therapeutic activity compared to solely relying on the EPR effect and other passive targeting strategies. Active targeting commonly involves attachment of a homing molecule onto either the therapeutic drug molecule, such as a doxorubicin-conjugate (Florent and Monneret, 2008) or onto a drug delivery system, such as doxorubicin-loaded liposomes (Shroff and Kokkoli, 2012; Petrenko and Jayanna, 2014). The homing molecule would then provide cell-specific accumulation of the therapeutic molecule specifically to the desired cells, thereby reducing major side effects and also reducing dose-limiting toxicities commonly seen with highly active molecules like doxorubicin.

Two broad mechanisms are commonly used to conjugate a targeting ligand into a liposomal drug delivery system: “pre-insertion” modifications and post-insertion modifications. “Pre-insertion” modifications consist of a covalent conjugation of the targeting ligand to one of the subcomponents of the drug delivery system before assembly of the nanoparticle. Attachment of a peptide/protein is commonly achieved by covalent modification of an activated lipid or polyethylene glycol (PEG)-lipid through either an amide conjugation or a disulfide/thioether conjugation (Wang et al., 2012). Following conjugation, the protein-lipid conjugates can then be used as one of the many assembly blocks used in a self-assembly mechanism to produce larger drug delivery systems, such as observed with liposomes (Lee et al., 2014). There are several advantages of attaching a ligand prior to nanoparticle assembly including: (1) highly controlled reaction conditions of ligand to a specific lipid, (2) more commonly available characterization methods available for quality control, and (3) a highly stable conjugation of the ligand to the lipid molecule. However, “pre-insertion” modifications often require a trained chemist to optimize reaction conditions/analyze products, result in moderate yields of the desired final product which then require purification, and can also degrade the functional activity of the targeting molecule depending on reaction conditions. Alternatively, post-insertion methods of nanomedicine modification involve attachment of a targeting ligand after assembly and drug loading. It has been reported that some liposomal nanomedicines are stable enough to perform an amide conjugation to an activated lipid exposed from the nanomedicine surface, however this still requires purification of final product before testing (Kung and Redemann, 1986). Another common method of post-insertion modification includes the use of a biotinylated lipid embedded in a lipid bilayer, which will then react almost irreversibly with a streptavidin-peptide conjugate or streptavidin-biotin-peptide conjugate (Loughrey et al., 1987). Another method of post-insertion modification includes the use of pVIII major coat proteins isolated from filamentous bacteriophage, such as M13 or fd, that display a targeting motif fused at the N-terminus of the protein (Petrenko and Jayanna, 2014). Post-insertion methods offer several advantages including rapid/simple modification reactions, milder reaction conditions that keep nanomedicines and ligands functionally intact, and stable non-covalent interactions between nanomedicines and the attached ligands. However, post-insertion methods can also cause nanomedicine instability or drug loss during modification that requires the optimization of reaction conditions to prevent undesired effects.

Filamentous bacteriophage of class Ff, including phage vectors such as M13, fd, and f1, are long cylindrical virions (~1 μm × 6.5 nm) structurally composed of a single-stranded DNA genome (<10 kbp) and 5 structural proteins (pIII, pVI, pVII, pVIII, and pIX) (Marvin et al., 2014). Each of the wild-type phage particles consist of 2700 copies of the pVIII major coat protein, which accounts for ~90% of the mass of each virion. The remaining 10% is derived from the phage's DNA genome and also a minor contribution from the four remaining structural proteins (<1%). Landscape phage display libraries of type f8, introduce a random peptide fusion into each of ~4000 domains of the pVIII major coat protein of the fd-tet vector to create a unique landscape across the surface of each phage clone (Smith, 1993; Petrenko et al., 1996; Kuzmicheva et al., 2009). Landscape phage libraries have been used extensively to generate ligands with specificity toward various cancer phenotypes (Jayanna et al., 2010a; Fagbohun et al., 2012; Bedi et al., 2014). The pVIII major coat proteins produced from these phage vectors have an 8- or 9-mer peptide fusion at the N-terminus and also contain an intrinsic membrane-spanning domain between residues 21–39 of the wild-type vector. This highly hydrophobic core serves to accumulate the pre-coat protein in the bacterial membrane during phage morphogenesis and also participates in formation of a highly stable phage particle to protect the genome from degradation. Filamentous phage particles and their isolated proteins show superior thermal stability while also maintaining the desired binding capacity compared to antibodies and also retain functional activity after exposure to harsh environments including relatively high temperatures (Brigati and Petrenko, 2005) acidic or alkaline solutions, moderate percentages of organic solvents (Olofsson et al., 1998) and protealytic enzyme treatment (Schwind et al., 1992) making filamentous phage and their pVIII major coat proteins ideal candidates for substitute antibodies in the targeted drug development (Petrenko and Smith, 2000; Petrenko, 2008). The intrinsic membrane domain of the pVIII major coat protein has provided an ideal platform to develop targeted nanomedicines by isolating the major coat protein and introducing the solubilized proteins into preformed, drug-loaded liposomes through spontaneous interactions of the hydrophobic core with the liposome bilayer (Jayanna et al., 2010b; Wang et al., 2010a; Petrenko and Jayanna, 2014). Several methods for solubilization and isolation of phage major coat protein have been studied including phenol extraction, several classes of detergents and a number of organic solvents. However, two critical factors that significantly effect the functional activity of the isolated protein include: (1) rapid aggregation rates of the protein due to the highly hydrophobic nature of the protein, and (2) an irreversible conformation change in secondary structure from α-helical to a β-sheet (Spruijt et al., 1989; Li et al., 2007; Siegel et al., 2015). Identification of a solvation procedure that allows high recovery of phage major coat protein in an α-helical conformation that would also limit damage to the membrane of pre-formed liposomes is ideal.

**Figure 1:**
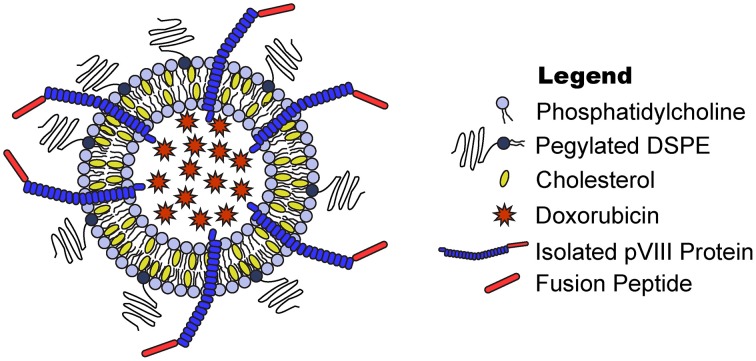
**Phage fusion protein-targeted nanoparticles**. Target-specific phage fusion protein selected from phage libraries can be introduced into drug-loaded PEGylated liposomes exploiting their intrinsic properties to spontaneously integrate into lipid bilayers.

One of the major limitations in ligand screening of targeted nanomedicines is due to highly complex reactions and optimizations required to prepare and purify a single targeted nanomedicine. In this study, we have developed a rapid protein isolation method to solubilize the pVIII major coat protein in high concentration, yield, and purity that also retains the desired functional activity. We show this isolated protein is then suitable to prepare targeted nanomedicines following a post-insertion modification strategy of preformed, drug-loaded liposomes (Figure 1). We hypothesized that optimization of ligand solubilization and insertion strategies would allow identification of cancer cell-specific ligands that increase the desired specific cytotoxic effect of liposomal nanomedicines in much higher throughput. We demonstrate the application of this technique for identifying ligands in a single combinatorial experiment combining novel cancer cell-specific ligands with a preformed liposomal doxorubicin core to generating 12 nanomedicines targeted with structurally unique ligands and subsequently screening for their anticancer activity *in vitro*. From this screening experiment, we were able to identify 8 novel targeting ligands able to significantly increase the therapeutic response of untargeted liposomal doxorubicin across two different cancer types.

## Materials and methods

### Reagents

Reagent grade or higher calcium chloride, chloroform, hydrochloric acid (HCl), 3-(4,5-dimethylthiazol-2-yl)-2,5-diphenyltetrazolium bromide (MTT), phenylmethylsulfonyl fluoride (PMSF), 2-propanol, proteinase K, and sodium dodecyl sulfate (SDS) were obtained from Sigma Aldrich (St. Louis, MO). Lipodox (PEGylated liposomal doxorubicin, composed of 1,2-distearoyl-sn-glycero-3-phosphocholine, cholesterol, 1,2-distearoyl-sn-glycero-3-phosphoethanolamine-N-[amino(polyethylene glycol)-2000] (ammonium salt) (DSPE- PEG2000) in molar ratio 56: 39: 5 containing ~2 mg/mL encapsulated doxorubicin) was obtained from SUN Pharmaceutical Ind. Ltd. (Gujaat, India).

### Cell culture

An established breast adenocarcinoma cell line, MCF-7 (ATCC, HTB-22™), and an established pancreatic adenocarcinoma cells line, PANC-1 (ATCC, CRL-1469™), were used throughout this study. All cell lines were purchased from the American Type Culture Collection (ATCC, Manassas, VA) as a frozen vial and maintained as described in the technical bulletins in a 37°C cell culture incubator with 5% CO_2_. Cells were maintained in the recommended basal medium supplemented with 10% defined fetal bovine serum (FBS) and 1% v/v 100X antibiotics/antimycotics (Ab/Am). Cells were tested for mycoplasma contamination using a commercial mycoplasma PCR detection kit (ATCC) and confirmed to be free of mycoplasma contamination following all experimental procedures. Eagle's Minimum Essential Medium (EMEM) and Dulbecco's Modified Eagle's Medium (DMEM) were obtained from ATCC. Hyclone defined FBS and Gibco 100X Ab/Am, a cocktail of 10,000 U/mL penicillin, 10,000 μg/mL streptomycin, and 25 μg/mL amphotericin B, were obtained from Thermo Fisher Scientific (Waltham, MA).

### Phage propagation and protein isolation in 2-propanol

All general phage handling, propagation, purification, titering, and DNA sequencing procedures have been described previously (Brigati et al., 2008; DeSantis et al., 2014). Phage clones isolated from landscape phage display libraries f8/8 and f8/9 were designated by the sequences of the foreign fusion peptides. Phage clones DMPGTVLP or VPTDTDYS were isolated from an *in vitro* biopanning of the f8/8 library over MCF-7 breast cancer cells; similarly, the phage clone VEEGGYIAA was isolated from an *in vitro* biopanning of the f8/9 library over MCF-7 breast cancer cells as described previously (Early Breast Cancer Trialists' Collaborative Group, 2005; Fagbohun et al., 2012; Riihimäki et al., 2012). Phage clones ANDVYLD, ANGRPSMT, GLNGRGDPD, or VNGRAEAP were isolated from the f8/8 library through *in vitro* biopanning with Calu-3 non-small cell lung cancer (NSCLC) cells, and phages DGQYLGSQ, DVRGDGLQ, EPSQSWSM, ETYNQPYL, or GSSEQLYL were isolated from the f8/8 library by *in vitro* biopanning with the PANC-1 pancreatic cancer cells, as described previously (Gabizon et al., 2003; Bedi et al., 2014).

Approximately 3 × 10^13^ phage particles dissolved in 1X TBS were transferred to a sterile 1.7 mL microcentrifuge tube followed by addition of 3 volumes of 100% 2-propanol and 30 μL of chloroform. Phage samples were vortexed vigorously to mix and shear intact phage particles into its primary components. Phage DNA was removed from the mixture by centrifuging at 13,000 ×g for 5 min at room temperature. The upper phase containing phage protein was transferred to a new sterile 1.7 mL microcentrifuge tube and stored at 4°C. Phage protein concentration was determined by measuring the UV/Vis absorbance of the solution at 280 nm with a NanoDrop. Absorbance values were converted to protein concentration, in mg/mL, using the appropriate 1 A(280) conversion factor as listed in Table 1.

**Table 1:** **Phage major coat protein (pVIII) properties**.

**Phage**	**MW (g/mol)**	**1 A(280) = X mg/mL**
ANDVYLD	5612.49	0.59
ANGRPSMT	5751.67	0.70
DGQYLGSQ	5786.62	0.58
DMPGTVLP	5747.72	0.70
DVRGDGLQ	5777.65	0.70
EPSQSWSM	5869.76	0.42
ETYNQPYL	5946.84	0.52
GLNGRGDPD	5703.57	0.69
GSSEQLYL	5815.70	0.58
VEEGGYIAA	5711.63	0.60
VNGRAEAP	5731.62	0.69
VPTDTDYS	5815.64	0.61

*Phage major coat protein (pVIII) properties were calculated following sequence analysis of the full length (55 amino acid) pVIII protein sequence using DNAStar Protean version 10.0.1. Phage are identified by their 8- or 9-mer fusion peptide sequence within the full length protein sequence of NH_2_-AXXXXXXXX[D/X]PAKAAFDSLQASATEYIGYAWAMVVVIVGATIGIKLFKKFTSKAS-COOH, where X can be any amino acid. Proteins with an 8-mer peptide fusion will contain a D in the 10th position of the pVIII fusion protein, while proteins with a 9-mer peptide fusion will have a random amino acid*.

### Characterization of isolated phage protein by SDS-PAGE

Two-fold serial dilutions of isolated phage protein in 1X Laemmli sample buffer were heated at 95°C for 1 h. A 10-fold dilution of the Precision Plus Protein WesternC Standards (Bio-Rad, Hercules, CA) was diluted in 1X Laemmli sample buffer and heated at 95°C for 10 min. Prepared samples were separated on a 4–20% Mini-PROTEAN TGX polyacrylamide gel (Bio-Rad, Hercules, CA) at a constant voltage of 100 V for 45 min. Following separation, the gel was fixed for 10 min in a methanol/acetic acid/water (v/v/v 50:10:40) fixing solution. Protein bands were labeled with a Colloidal Blue Staining Kit (Thermo Fisher Scientific, Waltham, MA) as described in the manufacturer's instructions. Briefly, the fixed gel was incubated in the prepared staining solution (stainer A/stainer B/methanol/water v/v/v/v 20:5:20:55) for 3 h at room temperature with gentle shaking. The staining solution was replaced with distilled water and destained for 11 h at room temperature with gentle shaking. Following destaining, bands were visualized using an EDAS Imaging Station (Eastman Kodak Co., Rochester, NY) equipped with a DC290 digital camera and a white light box. Images were captured and analyzed using Molecular Imaging Software (v. 4.0.3; Eastman Kodak Co., Rochester, NY).

### Preparation and characterization of phage protein-modified liposomal doxorubicin

#### Insertion of phage protein into liposomal doxorubicin (lipodox)

Phage protein-modified Lipodox was prepared at room temperature (20°C) by rapidly introducing isolated DMPGTVLP major coat protein into a preparation of Lipodox at a protein-to-lipid weight ratio of 1:200 as described previously (Fang et al., 2011; Wang et al., 2011). Briefly, isolated phage protein (2 μL) was added to 45 μL aliquots of Lipodox (90 μg doxorubicin) and mixed vigorously before addition of 1 volume of 1X TBS, pH 7.4 to the sample. Samples were incubated at 37°C overnight with gentle rotation. For screening assays, buffer was exchanged to dilute any residual 2-propanol by washing the liposomes in a 100 K NanoSep centrifugal filter device (Pall Co., Port Washington, NY) with 5 volumes of 1X PBS, pH 7.2 for 5 min. Samples were centrifuged at 14,000 ×g for 10 min and the flow through was removed. Liposomes were recovered from the retentate and analyzed as below.

#### Theoretical calculation of ligands per liposome

The number of lipids per liposomes was calculated based on the surface area of a unilamellar liposome with a bilayer thickness, h, of 5 nm; an average diameter, d, of 83 nm; and an average lipid headgroup area, a, of 0.5775 nm^2^ for the mixed lipid population according to previous reports (Pidgeon and Hunt, 1981) as discussed briefly below:

$$N_{Lipids}=\frac{\text{4}\pi }{a}\left[{\left(\frac{d}{2}\right)}^{2}+{\left(\frac{d}{2}-h\right)}^{2}\right]$$

The concentration of liposomes was subsequently calculated as follows, where the molar concentration of lipid in Lipodox, M, was 0.0216 M; and N_A_ is Avagadro's Number:

$$C_{Lipos}\left(Liposomes/mL\right)=\frac{M\times N_{A}}{N_{Lipids}\times 1000}$$

The number of ligands per liposome was calculated as follows, where N_ligand_ is the number of phage proteins calculated from the molar mass:

$$N_{Ligands/Liposome}=\frac{N_{Ligands}}{N_{Liposomes}}.$$

#### Purification of phage protein-modified lipodox by size exclusion chromatography

Protein modified Lipodox was purified by size exclusion chromatography (SEC) on a column (30 × 1 cm) packed with Superose 6 prep grade resin (GE Healthcare, Little Chalfont, UK) as described previously (Florent and Monneret, 2008; Jayanna et al., 2009). Liposomes were eluted with 10 mM Tris-HCl, pH 8.0 containing 0.2 mM EDTA at a flow rate of 0.25 mL/min. The elution profile was monitored by an Econo UV monitor (Bio-Rad, Hercules, CA) at an AUFS of 0.5 with fractions collected every 10 min (2.5 mL fractions). Fractions were stored at 4°C until further analysis.

#### Quantification of doxorubicin

Encapsulated doxorubicin was quantified by monitoring the absorbance at 492 nm following incubation with Triton X-100 (1% v/v final concentration) and comparing to a standard curve with known doxorubicin concentrations as described previously (Jayanna et al., 2010b; Shroff and Kokkoli, 2012; Petrenko and Jayanna, 2014).

#### Size distribution and zeta potential of liposomes

Liposome size distribution was determined using dynamic light scattering. Briefly, samples were diluted 100-fold with 1X PBS, pH 7.4 in a plastic sizing cuvette and analyzed on a ZetaSizer Nano ZS90 (Malvern Instruments Inc., Worcestershire, UK) maintained at 25°C with a scattering angle of 90° in triplicate. All liposome samples were found to be unimodal distributions, however samples were reanalyzed if multiple populations were identified. Size distributions are presented as the mean of triplicate Z-averages (nm) ± sample standard deviation.

Liposome zeta potential was determined by particle electrophoretic mobility as measured using laser Doppler electrophoresis and phase analysis light scattering. Briefly, samples were diluted 100-fold in 10 mM Tris-HCl, pH 8.0 with 0.2 mM EDTA in a plastic sizing cuvette and analyzed on a ZetaSizer Nano ZS90 with a Zeta Dip cell (Malvern Instruments Inc., Worcestershire, UK). Samples were maintained at 25°C during measurement and performed in triplicate. All recorded samples were equal in conductivity (± 0.01 of mean). Zeta potentials are presented as the mean of triplicate zeta potentials (mV) ± sample standard deviation.

#### SDS-PAGE and western blot of modified liposomes

Aliquots of each fraction following SEC were diluted with an equal volume of 2X Laemmli sample buffer and heated at 95°C for 1 h. Prepared samples were analyzed using a 4–20% Mini-PROTEAN TGX polyacrylamide gel at a constant voltage of 100 V for 45 min. Proteins separated by the SDS-PAGE were then transferred to a polyvinylidene fluoride (PVDF) membrane and blocked overnight with 1X protein-free PBS/0.05% Tween 20 blocking buffer. Membranes were probed with a rabbit polyclonal anti-fd IgG (1:5500 dilution, ~9 μg IgG) described previously (Smith et al., 1998; Wang et al., 2012) incubated with a biotin-SP-conjugated Affinipure goat anti-rabbit secondary IgG (1:30,000 dilution, ~1.3 μg IgG; Jackson ImmunoResearch Laboratories, Inc., West Grove, PA Cat # 111-065-003 RRID: AB_2337959) and with NeutraAvadin-Horseradish Peroxidase (HRP) (1:30,000 dilution) and visualized with West Pico substrate solution (Pierce, Rockford, IL). Membranes were imaged on a C-DiGit blot scanner (LI-COR, Inc., Lincoln, NE) and analyzed by densitometry with Image Studio Lite (v. 4.0; LI-COR, Inc.).

To ensure the N-terminus was exposed to the exterior environment of the liposome following phage protein modification, a proteinase K digestion assay was performed on modified Lipodox as described previously (Jayanna et al., 2010b; Lee et al., 2014). Briefly, an aliquot of protein-modified liposomes were treated with 1.25 μg of proteinase K containing 0.25 mM CaCl_2_ for 1 h at room temperature. The reaction was inhibited by the addition of PMSF to a final concentration 5 mM followed by incubation at room temperature for 5 min. Samples were analyzed by SDS-PAGE and Western blot as described above.

#### Phage protein orientation assay of modified liposomes by dot blot analysis

Samples were prepared as described above, except proteinase K reactions were performed overnight with gentle rotation at 37°C. Samples were then diluted 2-fold with distilled water and 1.0 μL of each sample were dotted onto a 0.2 μm nitrocellulose membrane (Bio-Rad Laboratories, Hercules, CA). Dots were allowed to dry overnight at room temperature. The resulting dots were probed with rabbit anti-Fd IgGs with specificity toward either the pVIII N-terminus (1:5000 dilution, ~9.24 μg rabbit anti-Fd IgG) or C-terminus (1:100 dilution, ~126 μg rabbit anti-Fd C-terminus IgG affinity purified). Membranes were then treated as above with a biotinylated-SP-conjugated goat anti-rabbit secondary IgG, NeutraAvadin-HRP and West Pico substrate before imaging with a C-DiGit blot scanner and quantified using Image Studio Lite.

#### Concentration of modified liposomes

Liposomes were concentrated by Amicon 100 k Molecular Weight Cut Off (MWCO) centrifugal concentration devices (EMD Millipore, Billerica, MA) as follows. Briefly, a 100 k MWCO concentrator was washed with 2 mL of 10 mM Tris-HCl, pH 8.0/0.2 mM EDTA running buffer. Samples were applied and centrifuged at 5000 × g in 15 min increments at 4°C until all samples were concentrated to a final volume of 0.5 mL. Doxorubicin liposomes found in the retentate were recovered and analyzed as above.

### Characterization of liposome modification by flow cytometry

Phage displaying the fusion peptide DMPGTVLP was propagated as above, however phage was dissolved in 1X PBS, pH 7.2 at the final recovery step. Phage proteins were labeled with an Alexa 488 Protein Labeling kit (Invitrogen, Waltham, MA) as described previously (Kung and Redemann, 1986; Fagbohun et al., 2012) and according to the manufacturer's instructions. Briefly, phage were added to a vial of reactive dye at room temperature and mixed for 1 h at room temperature. Unreacted dye was inactivated by addition of 1X TBS, 7.4 and removed by an overnight PEG precipitation of phage and solubilization in 1X TBS, pH 7.4. Phage protein was isolated in 2-propanol as described above and the degree of protein labeling was determined according to the manufacturer's instructions. Lipodox was modified with labeled protein as described above. Liposomes were analyzed on an Accuri C6 flow cytometer (BD Biosciences, Franklin Lakes, NJ) with an excitation wavelength of 488 nm and emission collected on three different channels (533/30, 585/40, and 670LP). The collection threshold was set to collect events greater than 10,000 on the forward scatter height (FSC-H) channel. After collection, a threshold to collect events greater than 1000 RFU on the 670LP height (FL3-H) channel was applied to reduce the background noise. A minimum of 100,000 events was collected per sample for analysis. Channels were compensated for channel spillover using single labeled controls. Labeled phage protein was monitored with the 533 nm emission filter channel and Lipodox was monitored on both 585 and 670 nm emission filter channels. Flow cytometry data were captured and analyzed using the Accuri C6 Software (ver. 1.0.264.21; BD Biosciences). All flow cytometry data files were annotated and deposited into the publically available flow cytometry data repository FlowRepository (Repository ID: FR-FCM-ZZJY) (Loughrey et al., 1987; Spidlen et al., 2012).

### Cell viability of cancer cells treated with phage protein-modified liposomes

Cell viability was determined by MTT assay as described previously (Bedi et al., 2014; Petrenko and Jayanna, 2014). Briefly, MCF-7 breast cancer cells were seeded at an initial density of 5 × 10^5^ cells per well in a 96-well cell culture-treated array plate and incubated at 37°C for 24 h. Lipodox samples (modified and unmodified) were diluted in EMEM containing 10% FBS and added to the cells for 24 h. For the screening assay, medium containing Lipodox was removed from the cells and replaced with an equal volume of fresh culture medium containing 10% FBS and incubated at 37°C for an additional 48 h before measuring viability. Following treatment, the medium was replaced with phenol red-free MEM containing 0.45 mg/mL MTT and incubated for 4 h at 37°C. After 4 h, 100 μL of 10% SDS in 0.01 N HCl was added to each of the wells and incubated at 37°C for an additional 4 h. The absorbance of the wells was measured at 570 nm using a Synergy H1 plate reader (BioTek, Winooski, VT). Blank wells containing only culture medium and MTT were subtracted from each sample. The percent viability was expressed as a ratio between the absorbance of treated cells by the average absorbance for a set of untreated cells.

### Quantification of cell-associated doxorubicin

Cell-associated doxorubicin was quantified as previously described (Bedi et al., 2014; Marvin et al., 2014) with minor modifications. Briefly, cells were plated at 5000 cells per well in a 96-well culture plate and incubated overnight in a 5% CO_2_ cell culture incubator at 37°C. Cells were then treated with 2 μg of doxorubicin of either (1) unmodified Lipodox or (2) DMPGTVLP-Lipodox for 4 different time periods (4, 8, 12, and 24 h). Cells were washed twice with 1X PBS, pH 7.2 before extracting cell-associated doxorubicin with 0.075 N HCl in 90% 2-propanol and incubating at 4°C overnight. Doxorubicin was determined by measuring the fluorescence (470 ex/590 em) with a Synergy H1 plate reader (BioTek, Winooski, VT) using untreated cells as a blank.

### Statistics

Data from all experiments are expressed as the mean ± sample standard deviation (SD). Differences between samples were determined using the Student's independent *t*-test or and alternative appropriately identified test listed in the text with a *P* < 0.05 as being statistically significant.

## Results

### Isolation of pVIII major coat protein in 2-propanol

**Figure 2:**
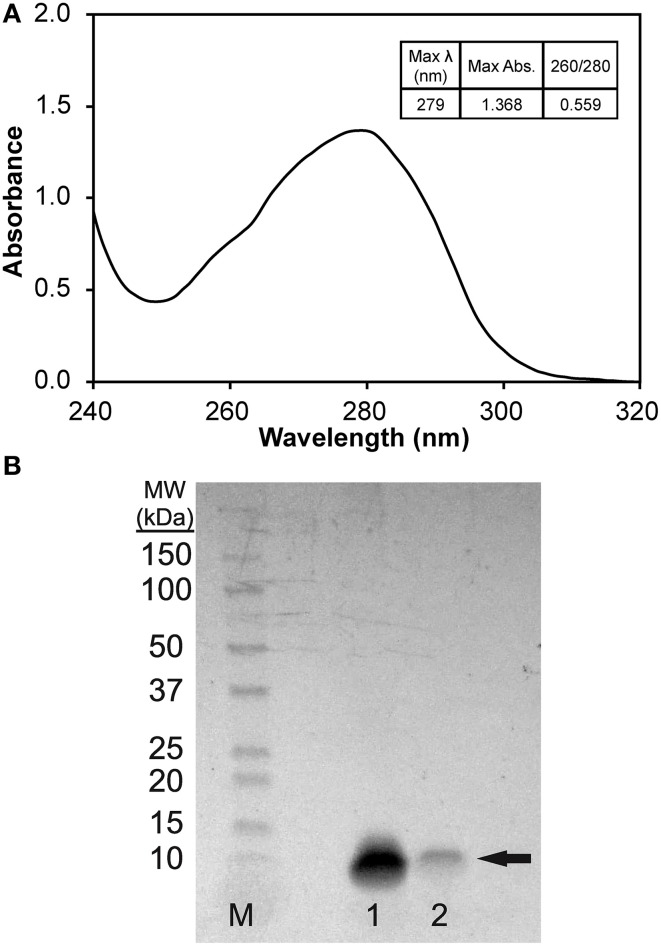
**(A)** UV/Vis spectrum (1 nm scan) of pVIII major coat protein solubilized in 75% v/v 2-propanol/1X TBS, pH 7.4 blanked with solution of 75% v/v 2-propanol/1X TBS, pH 7.4. **(B)** SDS-PAGE of pVIII major coat protein solubilized in 75% 2-propanol/1X TBS, pH 7.4 separated on a 4–20% polyacrylamide gel. Bands were identified by staining with a colloidal blue staining kit (Invitrogen) to reveal a single, low molecular weight band of ~7.5 kDa (as noted by the arrow) in samples 1 and 2. M (Marker)—1:10 dilution of Precision Plus Protein WesternC Standards (Bio-Rad), Sample 1–1.36 μg DMPGTVLP phage protein, Sample 2–0.27 μg DMPGTVLP phage protein.

To prepare solubilized phage major coat protein in 2-propanol, we added 3 volumes of 2-propanol to ~ 2.3 × 10^13^ virions of intact DMPGTVLP phage particles, which displays the full length, 55 amino acid pVIII major coat protein sequence of NH_2_-A**DMPGTVLP**DPAKAAFDSLQASATEYIGYAWAMVVVIVGATIGIKLFKKFTSKAS-COOH (designated shortly by the sequence of peptide fusion at the N-terminus; DMPGTVLP) and a small volume of chloroform to perturb the hydrophobic interactions between neighboring pVIII monomers (Manning et al., 1981; Smith, 1993; Petrenko et al., 1996; Kuzmicheva et al., 2009). After exposing intact phage to increased shear force by vortexing, phage ssDNA was released from the phage particles and precipitated in 75% 2-propanol. UV/Vis spectroscopy of the phage protein supernatant (Figure 2A) revealed a spectrum commonly observed for a pure protein sample with a corresponding 260/280 ratio of 0.559, which is close to the theoretical 260/280 ratio of 0.5 expected for pure pVIII major coat protein. Conversion of A280 into a corresponding mass revealed a quantitative recovery of the protein mass (~1 mg).

To test the purity of isolated phage major coat protein, serially diluted samples of phage protein were separated on a 4–20% polyacrylamide gel and stained with a colloidal Coomassie blue staining solution. The stained protein gel (Figure 2B) reveals a single, low molecular weight protein band at an apparent molecular weight of ~7.5 kDa, which corresponds to the monomeric-form of phage major coat protein (calculated molecular weight of 5.75 kDa, DNAStar Protean v. 10.0.1). Major coat protein, pVIII, was detectible down to ~200 ng of protein under these conditions with colloidal blue staining.

### Preparation and physicochemical characterization of phage protein-modified liposomal doxorubicin

**Figure 3:**
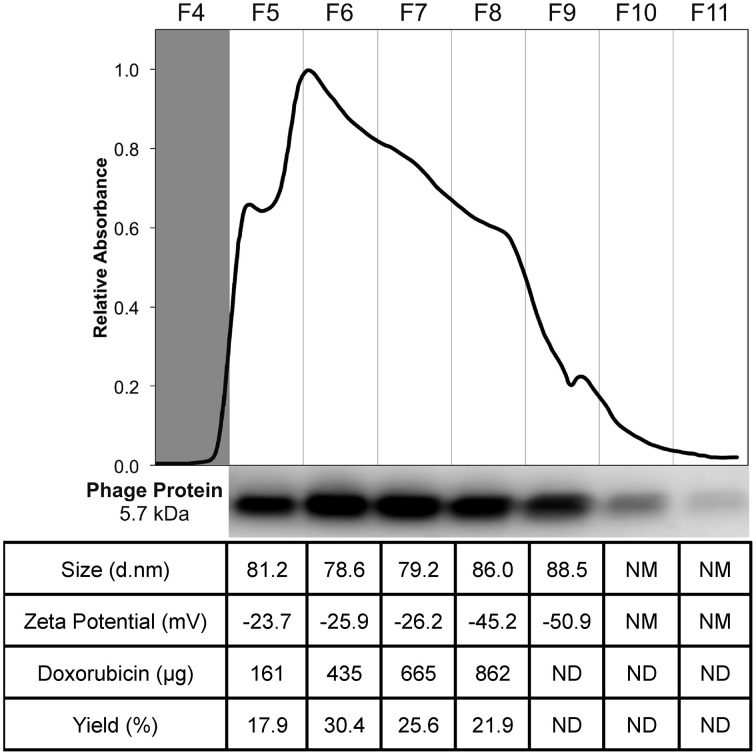
**Purification and characterization of DMPGTVLP-modified Lipodox**. Size exclusion chromatography of modified Lipodox on a Superose 6 column (30 × 1 cm), 2.5 mL/fractions 4–11 of the elution profile are shown. Western blot of recovered fractions probed with a polyclonal anti-fd rabbit primary IgG, followed by a biotinylated goat anti-rabbit secondary IgG and detected with NeutrAvadin-HRP and Pico West luminol substrate. Physicochemical characterization of recovered fractions for size distribution (d.nm), zeta potential (mV), doxorubicin recovery (μg), and percent doxorubicin recovery (%).

To prepare targeted Lipodox (pegylated liposomal doxorubicin, 2 mg/mL doxorubicin) preparations, 23.6 μg of isolated DMPGTVLP phage major coat protein was rapidly added to liposomes, composed of 4.73 mg of lipid and 0.9 mg of doxorubicin, in small aliquots to prevent dissolution of the liposomes. The residual 2-propanol and chloroform were diluted to a final concentration of 2.0 and 0.076% respectively, was shown previously to not cause an increased dissolution of liposomes (data not shown). Following incubation at 37°C overnight, samples were eluted through a size exclusion chromatography column packed with Superose 6 and the elution profile was monitored by relative absorbance with a UV/Vis monitor. The elution profile for DMPGTVLP-modified Lipodox (Figure 3) showed a single broad peak with 2 minor peaks at either side of the main peak (fractions 4 and 9). The broad peaks observed were also present in unmodified liposomes and is hypothesized to be due to the heterogeneous composition of the liposomes, however both minor peaks were excluded from further analysis.

To ensure phage protein was associated with the liposomes, a Western blot of each fraction (fractions 5–11) was performed with an anti-fd phage IgG that demonstrates specificity toward the N-terminus of the major coat protein. The blot revealed a single ~5.7 kDa molecular weight band with most of the phage protein associated with the liposomes found in fractions 6 and 7, however phage protein was also recovered in fractions 5–10 (Figure 3). Phage protein was not observed as free protein in the absence of liposomes. Fractions 5 through 8 were further characterized for recovery of doxorubicin and physical properties (size distribution and zeta potential) of the recovered liposomes. As shown in the summary table (Figure 3), 862 μg of doxorubicin (95.79% yield, 900 μg doxorubicin input) was recovered in the 4 fractions, which was significantly higher than previous post-insertion methods using cholate, which commonly resulted in 50–80% doxorubicin recovery. The size distribution of the resulting fractions showed a mean diameter of 81.25 ± 3.36 nm with an average PDI of 0.127 ± 0.029, which corresponds to a monodisperse population of liposomes with a low tendency for particle aggregation. The zeta potential of the fractions showed a mean zeta potential of −30.25 ± 10.0 mV with a mean conductivity of 0.020 ± 0.005 mS/cm, which suggests a generally negative surface charge of the liposomes in a solution of constant ionic strength and composition.

Since fractions 5 through 8 displayed similar composition and physical properties, they were concentrated with 100 K MWCO centrifugal concentration devices and analyzed again for final characterization properties. After concentration, 589 μg of doxorubicin (68.4% recovery, 862 μg doxorubicin input) was recovered; suggesting a large portion of doxorubicin was lost during concentration most likely due to doxorubicin sticking or passing through the membrane. Following concentration, DMPGTVLP-modified liposomes showed a mean diameter of 80.27 ± 1.30 with an average PDI of 0.104 ± 0.015, which shows no statistically significant change in size or aggregation due to concentration effects (two-tailed *t*-test, *P* = 0.605 and *P* = 0.196 respectively). DMPGTVLP-modified liposomes showed a significant 11.3% increase in size compared to unmodified Lipodox (72.07 nm; two-tailed *t*-test, *P* = 8.06 × 10^−3^), however this increase is negligible in the biological systems to be tested and is hypothesized to be due to swelling associated with major coat protein incorporation. After concentration, DMPGTVLP-modified liposomes showed a mean zeta potential of −19.87 ± 0.50, which again shows no statistically significant difference in zeta potential due to concentration effects (two-tailed *t*-test, *P* = 0.08). Modified liposomes showed a significant 11.4% decrease in zeta potential compared to unmodified Lipodox (−17.83 mV; two-tailed *t*-test, *P* = 4.2 × 10^−2^). We hypothesize that observed decrease might be due to the introduction of the fusion pVIII major coat protein, which contains a negatively charged N-terminus that is exposed to the environment following liposome modification and can influence the observed charge at the liposome surface.

**Figure 4:**
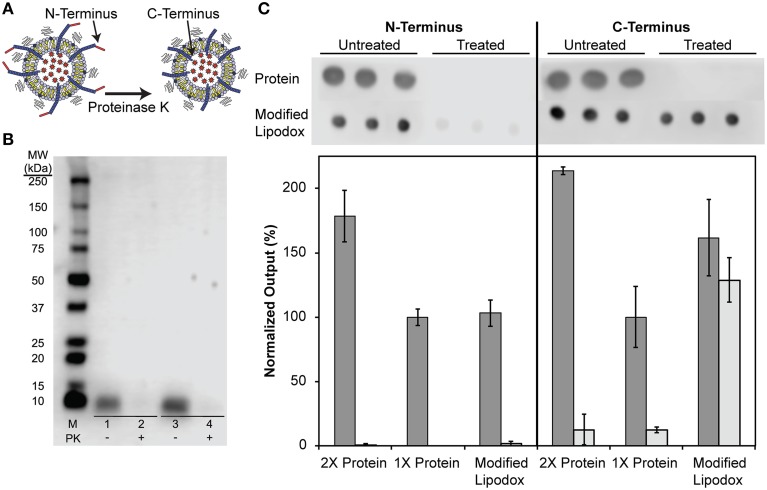
**(A)** Schematic of phage protein orientation assay, where it is expected that the N-terminus of the protein is exposed to proteinase K degradation while the C-terminus of the protein is protected from degradation by the lipid bilayer. **(B)** N-terminal orientation assay in DMPGTVLP-modified Lipodox. SDS-PAGE of concentrated DMPGTVLP-modified Lipodox followed by assay by Western Blot. M (Marker)—1:10 dilution of Precision Plus WesternC Standards, Samples 1 and 2–500 ng DMPGTVLP isolated protein, Samples 3 and 4—DMPGTVLP-modified Lipodox (~500 ng DMPGTVLP protein). Samples 1 and 3—untreated controls, Samples 2 and 4—Proteinase K (PK) treated samples. **(C)** Phage protein orientation assay by dot blot analysis with data quantified by densitometry and presented as the mean ± standard deviation of output signal normalized to 1X untreated protein (~375 ng protein). Untreated samples (dark bars) are compared to proteinase K treated samples (light bars) *N* = 3.

We next sought to determine if the N-terminus of the fusion protein, which contains the cancer cell-specific targeting domain, was exposed outside of the liposomal environment following concentration as previously shown (Jayanna et al., 2010a,b; Fagbohun et al., 2012; Bedi et al., 2014). As illustrated in Figure 4A, treatment of modified liposomes with proteinase K causes protein not protected by the liposome membrane to be hydrolyzed. To show that the N-terminus is exposed, a portion of modified liposomes was treated with proteinase K followed by SDS-PAGE and detection of the N-terminus of major coat protein with an anti-fd antibody. The resulting blot and densitometry of bands shows that isolated major coat protein can be detected as a single 5.7 kDa molecular weight band at the same protein mass used in a matched liposome modification (~500 ng of protein analyzed) and subsequently degraded to completion when treated with proteinase K under these conditions as expected (Figure 4B, lanes 1 and 2 respectively). After insertion into Lipodox, phage protein is detected in untreated samples and is absent following treatment with proteinase K (Figure 4B, lanes 3 and 4 respectively), suggesting that the N-terminus is exposed following modification and liposome concentration as expected.

As previously suggested, we hypothesized that the C-terminus of the phage major coat protein would be embedded in the hydrophobic membrane and protected from degradation by proteases. We therefore sought to determine the orientation of the C-terminus by a semi-quantitative dot blot assay. It was shown previously that degradation of the N-terminus produces a highly hydrophobic protein fragment that results in irreversible aggregation of monomers, causing visible smearing of bands in Western blots probed with a C-terminus specific antibody. To overcome this limitation and produce semi-quantitative estimates of the amount of each terminus following degradation, we optimized a dot blot assay and probed the resulting membranes with anti-fd IgGs with specificity to either the N- or C-terminus of the major coat protein (Figure 4C). Treatment of a 1X phage protein sample (~375 ng) with proteinase K resulted in complete degradation of the protein and was undetectable with both N- and C-terminal antibodies. To ensure reaction conditions were optimal for excess phage protein, a 2X phage protein sample (~750 ng) was digested under the same reaction conditions to again reveal complete degradation of the both N-terminus and C-terminus of the phage protein. Modified Lipodox (containing 375 ng phage protein) was digested with proteinase K using the same reaction conditions and probed with N-terminal and C-terminal specific antibodies. Probing the proteinase K treated Lipodox sample with the N-terminal antibody revealed complete degradation, confirming previously observed results obtained by Western blot (Figure 4B). Similarly, probing the same proteinase K treated Lipodox sample with a C-terminal specific antibody revealed no degradation of protein.

### Estimation of the degree of protein modification by flow cytometry

We further sought to determine an approximate degree of liposome modification by monitoring individual liposomes with flow cytometry. To estimate the modification efficiency of modified liposome, we fluorescently labeled intact phage particles with Alexa Fluor-488 and isolated the labeled protein as above. We have shown previously that intact phage can be modified with an amine-reactive Alexa Fluor-488 *N*-hydroxysuccinimide (NHS)-ester that reacts with the N-terminal amine and the exposed ε-amine of Lys-13 on pVIII major coat protein (Brigati and Petrenko, 2005; Fagbohun et al., 2012). The resulting degree of fluorescence labeling was calculated to be ~10% of the total phage protein mass, which is routinely achieved for phage after amine modification. Following isolation of pVIII protein, 25 μL of Lipodox (50 μg of doxorubicin) was modified with 14.4 μg of protein in which 10% of protein (1.4 μg) was labeled with Alexa Fluor-488.

**Figure 5:**
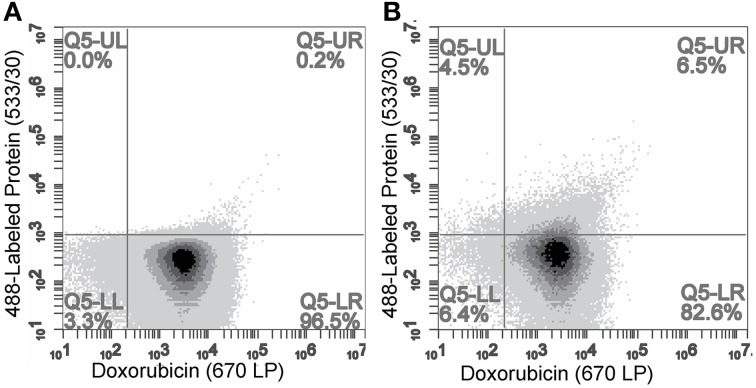
**Flow cytometry analysis of (A) unmodified Lipodox and (B) 488-labeled-DMPGTVLP-modified Lipodox**. Doxorubicin quantified on 670LP emission channel and 488-labeled phage protein quantified on 533/30 emission channel. Samples were excited using a 488 nm laser.

We hypothesized that these particles could then be quantified by flow cytometry with individual particles containing doxorubicin being identified by a relative fluorescence threshold of 1000 RFU set on the 670LP emission filter to reduce background noise. Unmodified Lipodox liposomes were analyzed across two channels (Em 585/40 and 670LP filters) to accurately identify liposomes from random background events. The Lipodox positive gates were identified on each channel from the samples displaying a linear correlation across both channels, identifying 95.6% of the sample population. Due to the broad emission spectrum of doxorubicin, the fluorescently labeled protein positive gate was identified at the maximum emission fluorescence of unmodified Lipodox using a 533/30-emission filter. The resulting gate also identified free-protein aggregates displaying high particle fluorescence. Analysis of unmodified Lipodox shows that 96.5% of the total population is found in the doxorubicin positive/protein negative quadrant (lower-right) with a minimal percentage in the doxorubicin/protein positive quadrant (upper-right) (Figure 5A). After liposome modification, there was a significant increase to 6.5% of the total population found in the doxorubicin/protein positive quadrant (upper-right) and a significant increase to 4.5% of the total population found in the doxorubicin negative/protein positive quadrant (upper-left) (Figure 5B). Since free protein was found to have high channel fluorescence, we suggest that the observed increase in the doxorubicin negative/protein positive quadrant (upper-left) is due to minor loss of encapsulated doxorubicin in modified liposomes below our doxorubicin positive gate. We therefore show a total detectible protein positive population of 11%.

### Cytotoxicity and uptake of DMPGTVLP-modified lipodox in breast cancer cells

**Figure 6:**
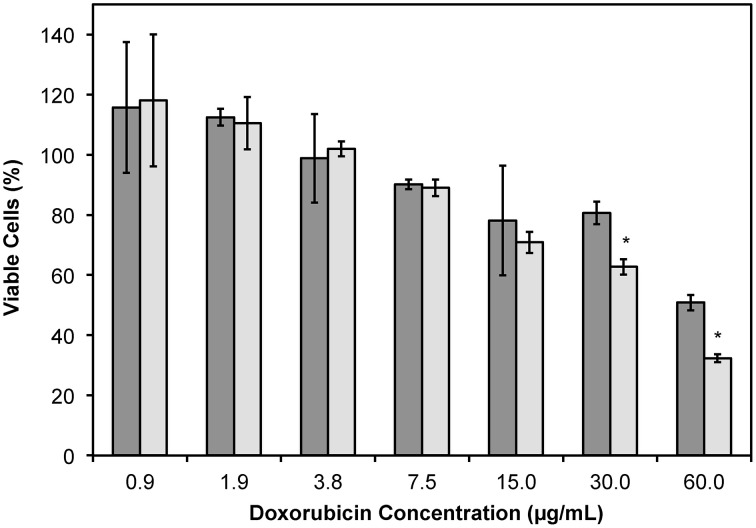
**MTT viability assay of MCF-7 cells treated with dilutions of Lipodox (dark bars) or DMPGTVLP-modified Lipodox (light bars) after 24 h of incubation**. Data are presented as the mean ± sample standard deviation of the percent viable fraction compared to untreated control cells, which were taken as 100% viable. *N* = 3; *P < 0.05, paired, two-tailed Student's *t*-test vs. unmodified Lipodox.

It was shown previously that DMPGTVLP-modified Lipodox, with modification by cholate solubilized phage protein, would significantly increase the toxicity in MCF-7 breast cancer cells *in vitro* and also *in vivo* (Olofsson et al., 1998; Wang et al., 2010a,b, 2014). We therefore sought to determine if liposome modification by phage protein solubilized in 2-propanol would produce similar results. MCF-7 cells were incubated with DMPGTVLP-modified Lipodox or unmodified Lipodox dilutions for 24 h before quantification of viable cells by MTT assay. DMPGTVLP-modified Lipodox produced a significant decrease in the number of viable cells compared to unmodified Lipodox samples under the same conditions in a dose dependent manner at higher doxorubicin concentrations of 30 μg/mL (62.7 ± 2.5 vs. 80.6 ± 3.7% viable; two tailed *t*-test, *P* = 8.11 × 10^−4^) and 60 μg/mL (32.3 ± 1.3 vs. 50.8 ± 2.5% viable; two tailed *t*-test, *P* = 4.97 × 10^−3^) as expected (Figure 6).

**Figure 7:**
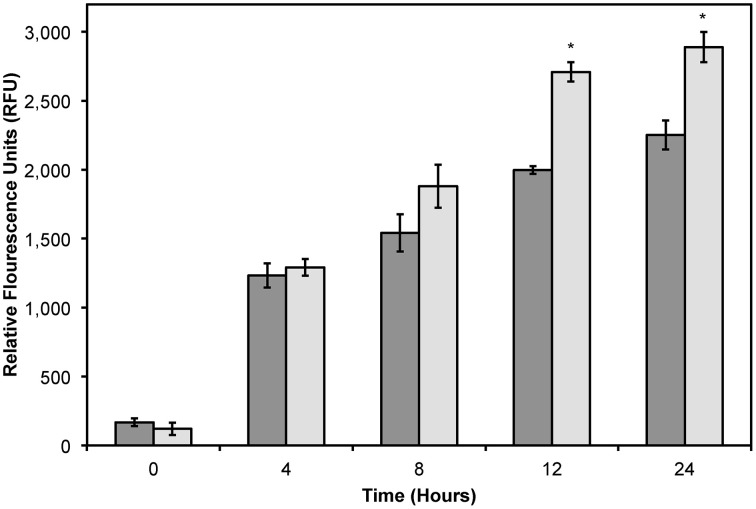
**Doxorubicin uptake assay in MCF-7 cells treated with 2 μg of Lipodox (dark bars) or 2 μg DMPGTVLP-modified Lipodox (light bars) over 24 h**. Data are presented as the mean ± sample standard deviation of the relative fluorescence of doxorubicin at an excitation wavelength of 470 nm and emission wavelength of 590 nm. *N* = 3; *P < 0.05, paired, two-tailed Student's *t*-test vs. unmodified Lipodox.

As shown previously, the increased cytotoxicity observed with DMPGTVLP-modified Lipodox is due to an increased uptake of targeted Lipodox in target cells compared to untargeted Lipodox. We therefore hypothesized that there would be a significant increase in intracellular doxorubicin over time in DMPGTVLP-modified Lipodox compared to unmodified Lipodox. We show over a 24-h time period, there is a significant increase in cell-associated doxorubicin in DMPGTVLP-modified Lipodox compared to the unmodified control Lipodox (Figure 7). After 12 h of incubation, there is a significant increase in fluorescence of ~710 RFU (two-tailed *t*-test, *P* = 1.82 × 10^−4^) corresponding to an increase of ~1.6 μg of total doxorubicin compared to unmodified (328.8 pg of increased doxorubicin/cell). Similarly, after 24 h of incubation, there is a significant increase in fluorescence of ~640 RFU (two-tailed *t*-test, *P* = 3.99 × 10^−3^) corresponding to an increase of ~1.5 μg of total doxorubicin compared to unmodified (294.9 pg of increased doxorubicin/cell). We therefore can confirm that modification of Lipodox by phage major coat protein in 2-propanol retains the previously observed increased cytotoxicity of targeted Lipodox via an increased delivery of doxorubicin to breast cancer cells.

### Screening of phage protein-modified lipodox

We next sought to screen a number of cancer cell-specific phage proteins isolated in 2-propanol to identify a panel of phage proteins that may enhance the specific toxicity of Lipodox toward cancer cells. We hypothesized that screening ligands in this manner would significantly increase the throughput of ligand identification for targeted drug delivery using a common Lipodox platform and varying the targeting ligand while retaining a common incorporation method. We initially screened 9 different ligands in MCF-7 breast cancer cells, which are commonly sensitive to doxorubicin. Next we screened an additional 5 ligands in PANC-1 pancreatic cancer cells, which are commonly reported to express a multiple drug resistance (MDR) phenotype and become resistant to doxorubicin treatment. Phage protein-modified Lipodox samples were prepared and characterized as above and summarized in Table 2 to identify preparation parameters for potential bias in the observed toxicity. As shown previously, all modified samples demonstrated a significant increase in mean size distribution (two-tailed *t*-test, *P* < 0.05). Five of the prepared samples (ANDVYLD, EPQSQWSM, VEEGGYIAA, DGQYLGSQ, and DVRGDGLQ) demonstrated a statistically significant change in zeta potential (two-tailed *t*-test, *P* < 0.05), however they were all within a biologically acceptable range. On average, most insertions produced high doxorubicin recovery rates (mean recovery 82.8 ± 20.4%) with no significant loss of doxorubicin following modification (mean loss 3.34 ± 2.02%). Modification with the protein ligand ANDVYLD caused significant loss of recovered doxorubicin (33.8%), however there was still sufficient material to complete the assay and all other parameters were similar to the other preparations.

**Table 2:** **Phage protein-modified Lipodox characterization**.

**Phage**	**Protein added (μg)**	**Recovered doxorubicin (%)**	**Free doxorubicin (%)**	**Size distribution (d.nm)**	**Zeta potential (mV)**	**IC_50_ (μg/mL)**
**ROUND 1—MCF-7 BREAST CANCER CELLS**						
ANDVYLD	0.41	33.84	1.61	81.75 ± 0.31^*^	−19.9 ± 0.1^*^	0.6
ANGRPSMT	0.86	109.02	1.72	80.53 ± 0.62^*^	−18.4 ± 1.4	0.5
DMPGTVLP	0.60	105.38	6.59	79.50 ± 1.44^*^	−18.3 ± 0.7	1.3
DVRGDGLQ	1.49	85.19	1.62	82.00 ± 0.66^*^	−19.0 ± 0.7	30.0
EPSQSWSM	1.24	104.46	2.83	80.47 ± 0.54^*^	−19.7 ± 0.3 ^*^	2.8
GLNGRGDPD	0.53	110.92	2.71	80.39 ± 1.40^*^	−18.7 ± 0.6	3.2
VEEGGYIAA	0.53	73.91	1.72	81.36 ± 1.28^*^	−21.3 ± 0.7 ^*^	2.6
VNGRAEAP	0.97	59.18	1.42	80.80 ± 1.49^*^	−18.4 ± 1.3	0.6
VPTDTDYS	1.38	77.99	1.61	80.74 ± 1.30^*^	−18.5 ± 0.4	3.3
No Protein	N/A	98.51	1.18	73.46 ± 0.49	−18.6 ± 0.4	13.5
**ROUND 2—PANC-1 PANCREATIC CANCER CELLS**						
DGQYLGSQ	1.58	75.75	4.88	89.11 ± 1.70^*^	−16.7 ± 0.5^*^	NC^a^
DVRGDGLQ	2.48	81.20	5.91	88.98 ± 0.50^*^	−16.7 ± 0.5^*^	NC^a^
EPSQSWSM	0.93	82.34	5.10	89.06 ± 0.91^*^	−17.7 ± 0.5	NC^a^
ETYNQPYL	0.50	80.37	6.37	90.72 ± 1.21^*^	−19.7 ± 0.8	NC^a^
GSSEQLYL	1.22	79.71	4.80	89.66 ± 1.07^*^	−17.4 ± 0.5	NC^a^

a *NC, Not calculated*;

* *p < 0.05, paired two-tailed Student's t-test vs. unmodified Lipodox (control)*.

**Figure 8:**
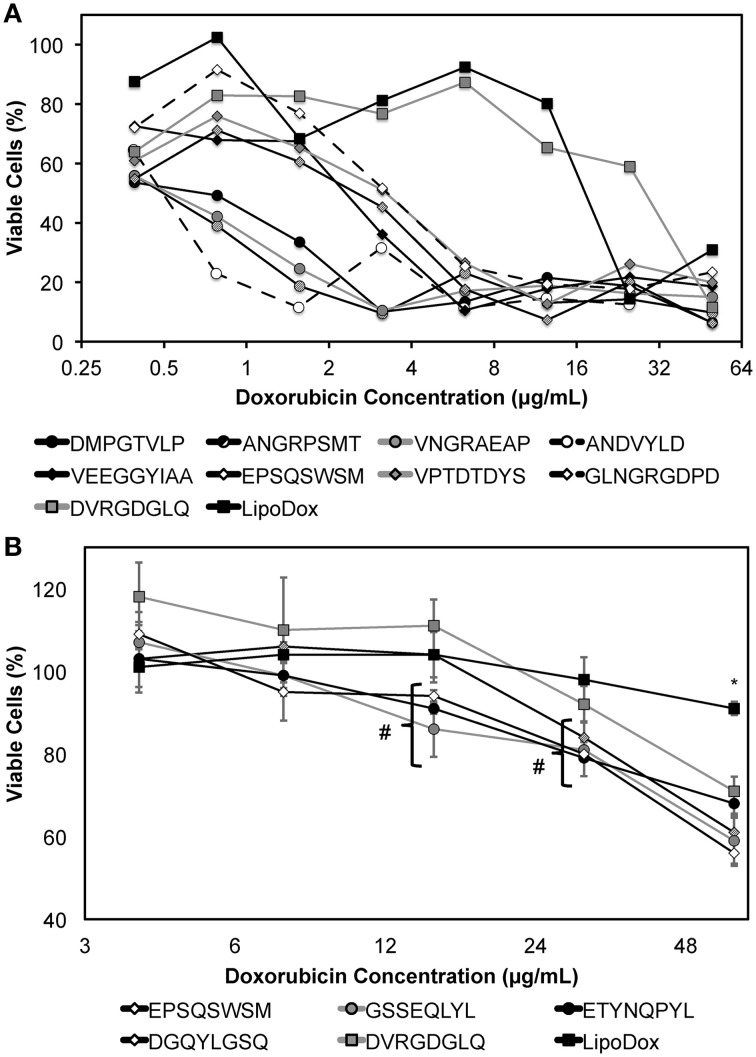
**MTT viability assay of (A) MCF-7 or (B) PANC-1 cells treated with dilutions of phage protein-modified Lipodox after 24 h of drug incubation followed by 48 h of drug washout**. Modified samples are identified by their displayed 8- or 9-mer fusion peptide sequence in the legend above. Data are presented as the mean ± sample standard deviation of the percent viable fraction compared to untreated control cells, which were taken as 100% viable. *N* = 3; *P < 0.001, paired, two-tailed Student's *t*-test vs. unmodified Lipodox; ^#^ *P* < 0.05, paired, two-tailed Student's *t*-test vs. unmodified Lipodox.

Following Lipodox modification and characterization, we hypothesized that targeting ligands specific for a certain cell type would increase the toxicity of Lipodox as suggested previously. MCF-7 breast cancer cells were treated with the 9 different phage protein-modified Lipodox and cell viability was measured 72 h after initial treatment. As shown in Figure 8A and from the calculated IC_50_ values in Table 2, there were a number of protein-modified Lipodox samples that significantly increased the toxicity profile of unmodified Lipodox, including DMPGTVLP as shown previously. The ligands screened stratified into three different groups covering a 100-fold change in IC_50_: (1) ligands showing no improvement from unmodified Lipodox (Figure 8A, squares), (2) ligands showing moderate improvement (Figure 8A, diamonds), and (3) ligands showing large improvements (Figure 8A, circles). As expected ligands with similar structural motifs grouped together, such as ANGRPSMT and VNGRAEAP, and produced similar IC_50_ values. Correlations between all factors in Table 2 compared to the IC_50_ were performed to identify parameters that might have influenced the toxicity profile apart from the targeting ligand and all parameters had a low to weak Pearson's R correlation (*R* < 0.3) suggesting the observed increase in toxicity were due to the cell-specific targeting ligands.

PANC-1 pancreatic cancer cells are shown to be resistant to doxorubicin by a MDR phenotype caused by overexpression of P-glycoprotein (Pgp) efflux pumps that prevent doxorubicin from accumulating within the cancer cells (Figure 8B, black square). However, we hypothesized that introduction of a cell-specific targeting ligand into Lipodox would be able to overcome the observed drug resistance. We showed previously that drug resistance could be overcome by addition of the ligand EPSQSWSM to Lipodox (Schwind et al., 1992; Bedi et al., 2014). We therefore sought to identify other ligands with specificity toward pancreatic cancer that would also significantly increase the toxicity of unmodified Lipodox in PANC-1 pancreatic cancer cells. Five ligands with specificity toward PANC-1 pancreatic cancer cells were inserted and screened as above with liposome characterization parameters identified in Table 2. As shown in Figure 8B, all ligands significantly increased the toxicity of unmodified Lipodox at high concentrations (two-tailed *t*-test, *P* < 0.001) and a subset of ligands [DGQYLGSQ, ETYNQPYL, and GSSEQLYL] showed a statistically significant increase in toxicity at doxorubicin concentrations of 12 and 24 μg/mL as well (two-tailed *t*-test, *P* < 0.05).

## Discussion

Although targeted nanomedicine delivery has demonstrated its efficiency in systemic cancer treatment in animal models and recently shown success in an ongoing Phase II clinical trial (Petrenko and Smith, 2000; Petrenko, 2008; Hrkach et al., 2012) identification of ideal targeting ligands for active tumor targeting remains a significant challenge. Many methods require multiple conjugation or purification steps before testing the functional activity of a ligand in a given nanomedicine scaffold. A number of these strategies are expensive, time consuming, require large batch volumes due to reactions that produce products with a low overall yield, or require modification of an identified ligand before conjugation. These limitations effectively reduce the ability to screen large numbers of ligands even within the same nanomedicine scaffold. Phage display has been commonly used as a source of novel ligands for various cancer related diagnostic or therapeutic markers. The most commonly used phage display libraries are performed with Ff class of filamentous bacteriophage vectors (including M13, fd, and f1) that displays a random peptide on the pIII minor coat protein, which supports a peptide insert of many different sizes (Smith and Petrenko, 1997; Jayanna et al., 2010b; Wang et al., 2010a; Petrenko and Jayanna, 2014). However, the pIII minor coat protein is only expressed in 5 copies at the infectious head of each phage particle, making it necessary to synthesize the identified cell-specific ligands separately from the phage and conjugate it to the nanoparticle carrier that may compromise the specific binding properties of the selected peptides. Previously, we have generated another type of display system—landscape phage in which the N-terminus of every copy of the pVIII major coat protein is modified with a randomized peptide fusion of 8 or 9 amino acids and have been used extensively to identify a number of cancer-specific ligands targeting a number of different cancer phenotypes (Romanov et al., 2001; Samoylova et al., 2003; Jayanna et al., 2010a; Fagbohun et al., 2012; Bedi et al., 2014). Since the pVIII major coat protein is expressed in 4000 copies per phage in these fd-tet-type vectors (Zacher et al., 1980) scaling of phage propagations up to 1 L scale can routinely produce protein yields up to 20 mg that can subsequently be purified from the bacteriophage genomic DNA using a number of standard techniques. Unlike the pIII minor coat protein, the recovered pVIII major coat protein can then be used directly in a number of drug delivery systems via a post-insertion modification of the nanomedicine scaffold using the inherent peptide domains designed naturally into the full-length coat protein as shown previously (Petrenko and Jayanna, 2014). Here we show a novel combinatorial extension of the phage display technology that allows testing for specific tumor cytotoxicity in a common nanomedicine core modified with different phage protein ligands in much higher screening throughput than traditional technologies to identify candidate ligands for further optimization.

In this study, we showed that the pVIII major coat protein could be isolated in 2-propanol to yield highly pure protein that was free from phage DNA. We hypothesize that the protein retains the α-helical secondary structure as suggested by the interaction of protein with liposomes and the solubility of the isolated protein. Under certain conditions and long storage conditions, the phage pVIII protein commonly adopts an irreversible β-sheet conformation, which causes the solubility of the isolated protein to decrease due to aggregation of large protein multimers (Spruijt et al., 1989). We do notice that extended storage of isolated phage protein in 2-propanol at room temperature will result in precipitated protein aggregates that remain insoluble in agreement with previous studies showing freshly prepared samples lose α-helical structure with time. Thus, freshly prepared phage proteins are recommended for all applications requiring α-helical protein structure, including modification of liposomes. The ability to retain the desired α-helical secondary structure following insertion into drug-loaded liposomes remains to be studied, however modified liposomes have been reported to maintain their targeting ability at least a month after preparation (Wang et al., 2014).

Following a similar insertion scheme as described previously using sodium cholate to solubilize pVIII proteins, we prepared a model drug delivery system consisting of isolated DMPGTVLP protein in 2-propanol inserted into preformed Lipodox liposomes. We then showed that liposomes were successfully modified with protein with significantly less loss of doxorubicin during modification while still retaining functional activity. As shown from a mock insertion of isolated DMPGTVLP phage protein into buffer, the complete loss of phage protein can be suggested to be due to the highly hydrophobic nature of the pVIII major coat protein causing any free protein to precipitate or adsorb to other hydrophobic materials. Similarly, as suggested by the partition coefficient of major coat protein for POPC (1-palmitoyl-2-oleoyl-sn-glycero-e-phosphocholine) lipid membranes reported to be 1.0 × 10^5^ M^−1^, it is hypothesized that 100% of the isolated protein will partition into the lipid bilayer primarily by hydrophobic interactions (Soekarjo et al., 1996).

**Figure 9:**
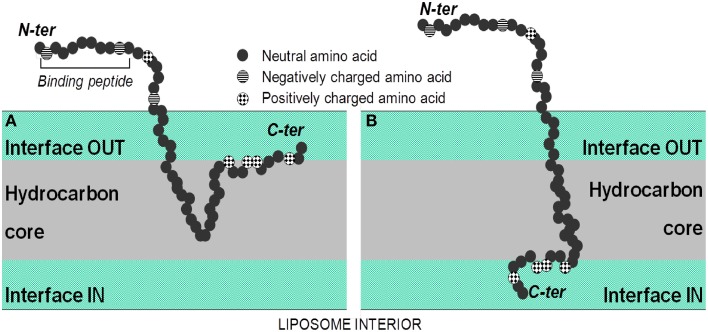
**Spontaneous insertion of the major coat protein into lipid membranes. (A)** In the first step, the isolated coat protein binds to the lipid membrane using electrostatic interactions of the C-terminus with negatively charged phosphate headgroups of lipids, followed by the insertion of the hydrophobic region of the protein into the hydrocarbon core of the lipid bilayer. **(B)** In the second step, the hydrophilic tail is released into the *trans* side of lipid bilayer through a process similar to cell-penetrating peptides. From Petrenko and Jayanna (2014).

In an attempt to identify the degree of protein labeling per liposome, we used semi-quantitative dot blotting and flow cytometry of intact liposomes to characterize protein-loading properties following Lipodox modification. From theoretical calculations based on mass of lipid/liposome and calculated molecular weights of phage proteins, it is estimated that there are ~50–100 ligands per liposome depending on the diameter of the liposomes used for modification. The data we obtained from dot blotting suggests there is no free protein in solution after liposome modification and we maintain a correct orientation of targeting protein with N-terminus exposed from the liposome and the C-terminus protected within the liposome core. Following degradation of phage protein-modified liposomes with Proteinase K, there was complete degradation of the N-terminus and no degradation of C-terminus. These data suggest that Proteinase K is sufficient to degrade all exposed proteins and 100% of the N-terminus is exposed from the liposome surface. We also estimate that 100% of the C-terminus is protected from degradation suggesting that the insertion of protein was complete and that no free phage protein is found in solution. We therefore show that the C-terminus of the pVIII major coat protein was able to translocate through the liposome membrane with high orientation specificity. We also show that following insertion of Alexa 488-labeled DMPGTVLP major coat protein into Lipodox, there was a significant shift of the liposome population toward the Alexa 488-positive gate. Only 11% of the liposome population showed a detectible increase in Alexa 488-positive labeling. Due to the low labeling efficiency (~10%) of the phage protein prior to insertion, we are unable to detect all ligand incorporations into the liposomes and can only resolve the most fluorescently intense liposomes receiving large numbers of Alexa 488-labeled phage proteins. Based on an estimate of 50 ligands/liposome, it can be expected that insertion of labeled proteins will follow a normal distribution with a mean of 5 labeled ligands/liposome. However, because of the poor resolution between the two populations, we can only observe the (11%) upper-tail of the normally distributed population which are the most bright. Optimization of fluorophore conjugation to the phage ligands or changing to a brighter fluorophore can increase the efficiency of this assay. It was also noted that there was a slight decrease in the doxorubicin mean channel fluorescence following modification (unmodified = 7929 RFU vs. modified = 5679 RFU in 670 LP-H channel) consistent with a slight loss in encapsulated doxorubicin. Free DMPGTVLP labeled protein appeared to aggregate heavily and form very bright particles, however in phage protein-modified Lipodox we observed only moderate increases in fluorescence. As we only see moderate increases in fluorescence, this suggests that there is no detectible free protein in solution with the modified Lipodox. This data is in agreement with previous reports on the completeness of insertion using different concentrations of isolate phage protein while maintaining a constant lipid mass up to a protein/lipid ratio of 1:200 (Soekarjo et al., 1996; Wang et al., 2011). We note that a significant limitation of this assay is the requirement for gating on a fluorescent population of liposomes. Since we are near the detection limits of the equipment, we found that empty liposomes, when performed with the same parameters, produce unreliable results as there is no precise method to identify liposomes from background noise during the measurement. We have seen that translocation through preformed lipid membranes is spontaneous (not requiring any host proteins), pH and temperature dependent. We propose that insertion of pVIII major coat protein is a two-stage process: (1) attachment of protein to the liposome surface, and (2) translocation of the C-terminus (Figure 9). The association of isolated pVIII major coat protein has been studied in detail previously, however the mechanism of C-terminal translocation has not been fully elucidated. We have previously proposed a mechanism in which the positively charged C-terminus is able to penetrate through a lipid bilayer (Petrenko and Jayanna, 2014) however detailed studies on the mechanism of translocation remain to be completed.

It remains to be seen whether there is a direct correlation between the strength of phage ligand binding to a target cell line and a subsequent increase in functional cytotoxicity activity *in vitro*. From our preliminary data, we find that sometimes weak binding phage clones perform better as targeting ligands in liposomal nanomedicines than strong binding phages. It has been suggested that low affinity ligands may be ideal for improved tumor penetration, as actively targeted drug delivery systems targeted by high affinity ligands are suggested to remain on the tumor periphery (Gray et al., 2013). For this reason, we used a functional screening assay of the final targeted nanomedicine in which new candidate ligands can be identified based on the desired increase in cytotoxicity rather than building an ideal drug delivery system based on optimization of individual components. For the functional screening assays, the drug exposure time was maintained at 24 h for all viability experiments to prevent excess drug leakage due to liposome degradation over the total measurement time. We have seen minimal doxorubicin leakage of phage protein-modified doxorubicin liposomes at 37°C in the presence of serum over the total test period (data not shown), however we sought to optimize screening conditions with alternative encapsulated drugs in mind displaying less carrier stability. We also increased the total incubation time of viability assays to a total of 72 h consisting of 24 h with phage protein-modified Lipodox samples plus an additional 48 h washout period in complete culture medium. The extended assay conditions allowed all cells, irrespective of origin, sufficient time to complete a normal cell cycle and thereby produce an increased toxicity profile in doxorubicin treated cells due to the inability of genomic DNA to replicate, resulting in a stalled cell cycle phenotype in the G_0_/G_1_ phase commonly observed with doxorubicin toxicity (Lukyanova et al., 2009). Given the broadly defined parameters, this screening assay can therefore be extended to a variety of cell types and also a number of drug classes employing different mechanisms of action. A limitation of this screening assay is the inherent nature of the MTT assay as a measure of mitochondrial succinate dehydrogenase activity in metabolically active cells rather than a cytotoxic event. However, as shown in previous work by us and other authors, doxorubicin will cause cell death by activation of caspase cascades leading to apoptosis (Wang et al., 2004). As we have not changed the properties of the cytotoxic drug and we have shown that phage proteins are non-toxic (Bedi et al., 2014) we don't expect significant changes in the primary mechanism of cell death, only an increase in intracellular delivery of cytotoxic drug. Since we have shown previously that the MTT assay is a good indicator of cell death in this system and death can be confirmed by visual observations, the reduction in cell viability as determined by the MTT assay would be sufficient for identification of ligands in a screening assay.

From our screening experiment in MCF-7 cells, we were able to identify a previously validated breast cancer cell-specific protein DMPGTVLP that interacts with cell surface expressed nucleolin and increases the therapeutic effect of liposomal doxorubicin *in vitro* and in *vivo* by increasing the specific delivery of doxorubicin to MCF-7 cell nuclei (Wang et al., 2010a, 2014; Fagbohun et al., 2012). Liposomes modified with a phage fusion protein displaying the peptide ANDVYLD showed the greatest increase in toxicity with a calculated IC_50_ of 0.6 μg/mL in MCF-7 cells. We also identified two peptides containing a positionally constrained **NGR** motif (A**NGR**PSMT and V**NGR**AEAP) that showed similar toxicity profiles (average IC_50_ = 0.55 μg/mL) as expected. Surprisingly, phage VNGRAEAP did not show binding to MCF-7 cells in our previous binding assays, however this limitation may be due to a short binding assay duration (1 h) compared to the duration of the cytotoxicity assay (24 h) or due to a difference in primary uptake mechanism between the two phage as shown previously (Gillespie et al. Submitted). A similar motif containing a positionally different **NGR** motif (GL**NGR**GDPD) showed improvement in toxicity to 3.2 μg/mL, however it was unable to reach the same toxicity of the other motifs. This may be due to a difference in the NGR motif availability to the receptor or an effect of peptide affinity. Inclusion of a pancreatic cancer-specific ligand (DVRGDLQ) resulted in no significant increase in toxicity suggesting the specificity of ligands to increase doxorubicin delivery specifically to the target cells and is not a result of modification. Screening the DVRGDLQ peptide in pancreatic cancer cells produced an increase in cytotoxicity compared to unmodified Lipodox. Interestingly, a subset of ligands with similar motifs (DGQYLGSQ, ETYNQPYL, and GSSEQLYL) grouped together to produce similar cytotoxicity profiles. Comparison of a shared **D**XXXX**G**X**D** motif showed that there was a significant difference between DVRGDGLQ and GQYLGSQ ligands at higher concentrations (two-tailed *t*-test, *P* < 0.01) with the DGQYLGSQ ligand producing greater toxicity suggesting minimal involvement of the **D**XXXX**G**X**D** motif. Comparison of the **YL** motif found in different positions of the three ligands showed no statistical differences between the three ligands (two-tailed *t*-test, *P* > 0.05), however all three showed significant differences compared to unmodified. These data therefore suggest the **YL** motif, irrespective of position, will cause an increase in toxicity. No putative receptor has been suggested for any of the three ligands at this time, however it is interesting to note the number of essential amino acids (Y, L, W, and V) present in the identified ligands that are typically underrepresented in our phage display library (Kuzmicheva et al., 2009). The presented data demonstrate the use of phage display technology along with a combinatorial screening strategy to significantly enhance the screening throughput of targeted nanomedicines that may considerably accelerate progress in actively targeted drug development.

## Author contributions

JG, DB, AG, and AP designed and performed experiments, contributed to the interpretation of results, wrote the manuscript and approved the final version for publication; VP designed the research strategy and experiments, contributed to the interpretation of results, wrote the manuscript and approved the final version for publication.

### Conflict of interest statement

The authors declare that the research was conducted in the absence of any commercial or financial relationships that could be construed as a potential conflict of interest.
